# Association between vitamin D and glycaemic parameters in a multi-ethnic cohort of postmenopausal women with type 2 diabetes in Saudi Arabia

**DOI:** 10.1186/s12902-021-00825-3

**Published:** 2021-08-12

**Authors:** Shatha Alharazy, Eman Alissa, Susan Lanham-New, Muhammad Imran Naseer, Adeel G. Chaudhary, M Denise Robertson

**Affiliations:** 1 Department of Physiology, Faculty of Medicine, King Abdulaziz University, Jeddah, Saudi Arabia; 2 Department of Clinical Biochemistry, Faculty of Medicine, King Abdulaziz University, Jeddah, Saudi Arabia; 3 Department of Nutritional Sciences, Faculty of Health and Medical Sciences, University of Surrey, Guildford, UK; 4 Center of Excellence in Genomic Medicine Research, King Abdulaziz University, 21589 Jeddah, Saudi Arabia; 5 Department of Medical Laboratory Technology, Faculty of Applied Medical Sciences, King Abdulaziz University, 21589 Jeddah, Saudi Arabia; 6 Centre for Innovation in Personalized Medicine, King Abdulaziz University, Jeddah, Saudi Arabia

**Keywords:** Postmenopausal, Vitamin D, Deficiency, Type 2 diabetes, Insulin Sensitivity, Insulin resistance, Saudi Arabia

## Abstract

**Background:**

The relationship between vitamin D (VitD) and insulin sensitivity and secretion in type 2 diabetes mellitus (T2D) has been shown to be different amongst different ethnic populations. In Saudi Arabia, where both T2D and VitD deficiency are highly prevalent health concerns, little is known about the relationship between VitD, insulin sensitivity, resistance and the relative importance of ethnicity. Our primary aim in this study was to investigate influence of ethnicity on VitD association with glycaemic profile and to measures of obesity as a secondary outcome, among multiethnic postmenopausal women with T2D in Saudi Arabia.

**Methods:**

A cross-sectional study was conducted at King Fahad Medical Research Center, King Abdulaziz University, Jeddah, Saudi Arabia. Postmenopausal females (*n* = 173, age ≥ 50 years) with T2D were randomly selected in this study. Anthropometric measures and fasting blood samples were obtained for all study participants. Several biochemical parameters were measured including 25-hydroxyvitamin D (25(OH)D), glycosylated hemoglobin (HbA1c), insulin, glucose and c-peptide. Surrogate markers for insulin resistance were calculated using Homeostasis Model Assessment 2 for insulin resistance and beta cell activity (HOMA2-IR, HOMA2-β).

**Results:**

Overall, 25(OH)D was inversely associated with fasting glucose (*r*=-0.165, *P* = 0.037), insulin (*r*=-0.184, *P* = 0.02), C-peptide (*r*=-0.19, *P* = 0.015) and HOMA2- IR C-peptide (*r*=-0.23, *P* = 0.004). Additionally, serum 25 (OH)D showed a negative correlation with body weight (*r*=-0.173 *P* = 0.028), waist and hip circumferences (*r*=-0.167, *P* = 0.033; *r*=-0.22, *P* = 0.004 respectively) but not with body mass index (BMI) or waist hip ratio (WHR).

In the white ethnic group but not in black or Asian population groups, 25(OH)D level was also associated with only serum fasting C-peptide and HOMA2-IR C-peptide and BMI (*P* < 0.05).

**Conclusions:**

Insulin resistance and obesity were associated with VitD status in T2D in this cohort. Our findings also suggest that these VitD associations in women from white ethnic background are different than in those from black/Asian ethnic backgrounds. Whether VitD supplements are able to improve either obesity and/or insulin sensitivity should be further investigated in different ethnic population groups.

## Background

Vitamin D (VitD) has a pivotal role in the regulation of calcium (Ca) concentrations in blood through its influence on intestinal absorption and bone metabolism and through its interaction with calciotropic hormones [1]. The influence of VitD on extra-skeletal tissue is gaining increasing prominence in the literature, thought to contribute to insulin resistance, pathology of pancreatic β -cell and systemic inflammation and ultimately type 2 diabetes mellitus (T2D) risk [2–7]. It is proposed that VitD can influence the progression and control of T2D either directly by binding to its own receptor (VitD receptor) on β-cells of the pancreas or indirectly by regulating extracellular Ca or Ca influx to pancreatic β-cells [8, 9].

VitD deficiency/insufficiency, which is assessed by circulating blood 25-hydroxyvitamin D 25(OH)D concentration, is one of the most globally widespread health concerns among postmenopausal women [10] and has been suspected as a risk factor for T2D in Europeans, African-Americans and south Asians [4–7]. It has also been reported that an inverse relationship exists between VitD status and risk of T2D and metabolic syndrome [11].

In several observational studies, VitD deficiency has been linked to insulin sensitivity and secretion which can both be impaired in T2D; however the role of ethnicity has not been fully examined [2, 12, 13]. A large cross-sectional study [14] in the US revealed that the Homeostatic Model Assessment of Insulin Resistance (HOMA-IR) and β-cell secretion (HOMA-%β) showed no association with serum 25(OH)D level in non-Hispanic black individuals. However, the same study revealed a correlation between 25(OH)D levels and HOMA-IR in non-Hispanic whites and Mexican Americans. This finding can be interpreted as due to a lower responsiveness to VitD and parathyroid hormone (PTH) in blacks in comparison to whites [14].

Saudi Arabia has a multi-ethnic population and both T2D and VitD deficiency are highly prevalent and of widespread concerns [15, 16].

There are few data in the literature from Saudi Arabia concerning the association/relationship between VitD, insulin sensitivity and resistance with consideration of ethnicity. The aim of this study therefore was to investigate the effects of ethnicity on VitD (25(OH)D) associations with insulin sensitivity, diabetic control and measures of obesity in postmenopausal women in Saudi Arabia with T2D; to target prospectively the ethnic group with stronger VitD associations for VitD dosing treatment.

## Methods

### Study design and recruitment

This cross-sectional study was conducted on 173 postmenopausal women with T2D, aged between 50 and 87 years, living in the western region of Saudi Arabia (Jeddah). We assessed VitD status (25(OH)D) in the participants and its association with: (1) glycaemic parameters (HbA1c, fasting glucose, fasting insulin, fasting C-peptide and insulin sensitivity indices); (2) bone related parameters (intact PTH, Ca, albumin, phosphorus (PO4) and magnesium (Mg)); (3) anthropometric measures (weight, height, waist hip ratio (WHR) and BMI); (4) lifestyle factors (physical activity, smoking, dietary VitD intake, veiling and sun exposure); (5) Socio-demographic factors including skin tone and ethnicity.

Subjects for this study were sequentially recruited from seven primary health care centers (PHCCs) distributed in Jeddah (a PHCC from each of the seven geographical sectors of Jeddah area was chosen to represent a randomly selected adult population). A multi-stage sampling technique was implemented. In stage 1, one PHCC was chosen from each of the seven sectors of the Jeddah area. In stage 2, random selection of samples was conducted from the selected PHCCs to select female files of the registered population. In stage 3, all women in the selected age group (≥ 50 years) among selected files were contacted for possible recruitment to the study based on the predefined criteria of inclusion. The number of women randomly selected from each center was proportionally identified according to the number of the registered women in each center. Subjects willing to participate in this study were referred to a clinic at the Centre of Excellence for Osteoporosis Research (CEOR) in King Fahd Medical Research Centre (KFMRC), King Abdulaziz University (KAU), Jeddah. Each participant provided written informed consent relating to participation in this study. Following the ethical standards in Declaration of Helsinki, ethical approval of this study was obtained from the Research Ethics Committee, the Faculty of Medicine, KAU (ref no.179 − 16, Oct/2017).

**Fig. 1:**
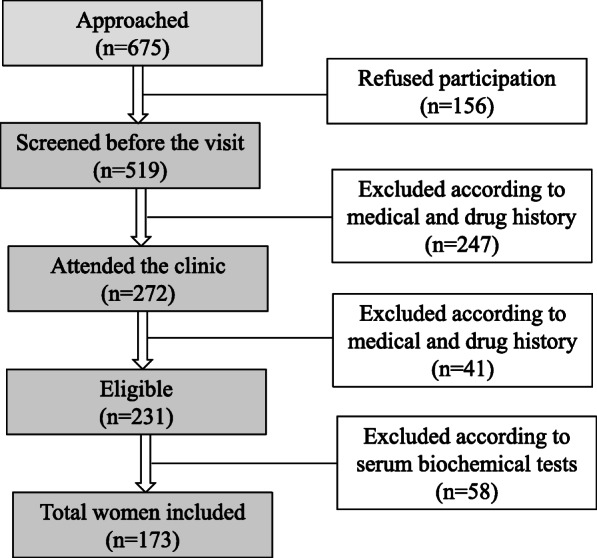
Flow chart of the study postmenopausal participants with T2D

The recruitment and selection of patients was based on specific inclusion and exclusion criteria. Inclusion criteria included postmenopausal women: Last menstrual period (LMP) ≥ 1year and follicular stimulating hormone (FSH) > 15 IU/L), previously diagnosed with T2D according to the criteria of the American Diabetes Association ( HbA1c ≥ 48 mmol/mol or fasting plasma glucose ≥ 7 mmol/L) [17]. Women with history of chronic liver or renal disease, cancer, malabsorption syndrome, rheumatoid arthritis, hyperthyroidism, other endocrinal disorders that might affect bone (e.g. Hyperparathyroidism) or history of intake of medications with possible effects on VitD (e.g. VitD supplements, glucocorticoids and anticonvulsants) were excluded from the study. Sample size was calculated as follows: The total number of females aged between 50 and 79 years living in western region of Saudi Arabia is approx. 430,739, according to the latest demographic survey of the general Saudi authority for statistics (https://www.stats.gov.sa/en). The prevalence of VitD insufficiency (serum 25(OH)D < 20 ng/ml) in Saudi Arabia is 87.8 % [18]. The maximum expected prevalence of VitD insufficiency and deficiency is 90 %. The level of confidence is 95 % with study power of 0.80. Accordingly, the calculated sample size was 150 females, determined by a validated sample size determinant program (Epi-Info, version 6, GA, USA). The sample size was increased by 15 % to guard against drop out and to increase the validity of the study. Following multiple stages of exclusion (Fig. 1), a sample size of 173 was included in this study.

Initially, all participants answered a questionnaire (completed by the researcher), which requested information including socio-demographic factors, dietary VitD intake (semi-quantitative Food Frequency Questionnaire (SFFQ) [19], lifestyle history including smoking habits and physical activity, medical history, menstrual history and drug history. Each participant underwent anthropometric and blood pressure measurements.

Skin tone was recorded for each participant based on the Fitzpatrick skin tone classification [20]. Duration (number of hours) of weekly exposure to outdoor sunlight in the last month was noted in the participants’ questionnaires as well as the use of sunscreen. Due to cultural or religious reasons, most women in Saudi Arabia, especially the elderly, wear a veil and cape. Women participating in the study who cover their head and body, with face and hands exposed were considered as partially covered, while participating women covering their whole body and face, with only the eyes and hands exposed were considered as totally covered.

The daily VitD intake from food was estimated using a semi-quantitative Food Frequency Questionnaire (SFFQ). The SFFQ used in the study was adapted from a validated SFFQ in Saudi Arabia [19]. Items included the most commonly VitD rich food consumed in the region; salmon, tuna, sardines, milk, laban (buttermilk), yogurt, egg and beef liver. The frequency of this food intake was expressed as number of servings per day/week/month. The daily VitD intake in IU was then calculated and compared to their estimated average requirement (EAR) (600–800 IU/day based on the IOM recommendation for women aged 50 y and over [21].

### Serum measurements of 25(OH)D and other hormones

Serum 25(OH)D and intact PTH levels were measured by direct competitive chemiluminescence immunoassay (CLIA), using a LIASON auto-analyzer (DiaSorin Inc, Stillwater, MN, USA). The intra and inter-assay coefficient of variations (CV) of serum samples were < 8 %. VitD deficiency was defined based on Institute of Medicine (IOM) guidelines [21] as the 25(OH) D level below 12 ng/ml (< 30 nmol/l) and VitD insufficiency as the 25(OH)D level of 12–19 ng/ml (30–49 nmol/l), and VitD sufficiency between 20 and 50 ng/ml (50–125 nmol/L). FSH and Thyroid function test (TFT) including thyroid-Stimulating Hormone (TSH), free thyroxin (T3) and free triiodothyronine (T4) were measured in serum by immunoassays, using VITROS ECiQ (Ortho-Clinical Diagnostics Inc., Rochester, NY, USA) to exclude any women with hyperthyroidism or not postmenopausal.

### Serum measurements of liver enzymes, renal function, high-sensitivity C-reactive protein, lipid and bone profile

Liver enzymes (including Aspartate Aminotransferase (AST), Alanine Aminotransferase (ALT), and Alkaline Phosphatase (ALP)), creatinine, total cholesterol, direct high density lipoprotein cholesterol (HDL-C), triglycerides, low density lipoprotein cholesterol (LDL-C), very low density lipoprotein cholesterol (VLDL-C) albumin, Ca, PO4 and Mg) were all measured in serum by reflectance spectrophotometry, employing the colorimetric method using a VITROS 250 Clinical Chemistry Auto-analyzer (Ortho-Clinical Diagnostics Inc., Rochester, NY, USA). The intra and inter-assay CV of serum samples were 4.1 and 4.5 % respectively. LDL-C concentrations in serum were directly calculated by the analyzer, based on standardized calculations (Friedewald equation) [22] dependent on the level of total cholesterol, direct HDL-C and triglyceride measured by the same analyzer. VLDL-C serum levels were estimated by dividing the triglyceride by 2.2. Subjects with high liver enzymes were excluded (the normal clinical level of serum being AST ˂45 U/L; ALT ˂50 U/L and ALP between 80 and 280 U/L). Samples with creatinine levels higher than normal were excluded (i.e. a normal level of serum creatinine in females ˂105µmol/L).

High sensitive C-reactive protein (hs-CRP) was measured in serum by immunoassay, using a VITROS 5,1 FS chemistry auto-analyzer (Ortho-Clinical Diagnostics Inc., Rochester, NY, USA). The intra-assay and inter-assay CV of serum samples were 3.5 and 4 % respectively.

### Measurements of glycaemic control parameters

Glycosylated hemoglobin (HbA1c) was determined using a VITROS 5,1 FS chemistry auto-analyzer (Ortho-Clinical Diagnostics Inc., Rochester, NY, USA). Fasting glucose in serum was measured by means of a colorimetric method, using a VITROS 250 Clinical Chemistry Auto-analyzer (Ortho-Clinical Diagnostics Inc., Rochester, NY, USA). The intra and inter-assay CV for HbA1c and fasting glucose samples were both < 5 %. Insulin and c-peptide (a consequent product produced when insulin is secreted) were measured in serum by a sandwich CLIA using a LIAISON autoanalyzer (DiaSorin Inc, Stillwater, MN, USA). The intra and inter-assay CV for insulin and C-peptide serum samples were both < 6 %. Fasting insulin and Homeostasis Model Assessment 2 (HOMA2) [23] were measured for all women, with the exception of those on insulin therapy, due to the influence of insulin intake on these measures.

HOMA2 was used to estimate insulin resistance and β-cell function. HOMA2 indices [24] (HOMA2-IR and HOMA2-%β) were calculated from fasting glucose, fasting insulin and fasting c-peptide in a steady-state condition (fasting glucose: 3–25 mmol/L, fasting insulin: 2.88–43.16 mIU/L and fasting c-peptide: 0.6–10.5 µU/ml) using an updated computer HOMA calculator software (version 2.2.3) issued by University of Oxford Diabetes Trials Unit, available at https://www.dtu.ox.ac.uk/homacalculator/ .

### Statistical analysis

The statistical analysis was performed using SPSS program (v.20 SPSS Chicago Inc). Normality of data was tested by Kolmogorov-Smirnov test. All numerical parametric results were expressed as means ± SD, while numerical non-parametric results were presented as median (IQR). Descriptive results were expressed as a percentage of the total sample number. Correlations between different parameters were obtained using Pearson correlation for normally distributed data and Spearman correlation for non-normally distributed data. The non-parametric test, Kruskal-Wallis H test, was used based on non-normally distributed data to compare between groups. A probability value ≤ 0.05 was considered statistically significant. Multiple linear regression analysis (stepwise) was used for independent variables that showed significant correlations at the bivariate level (*P* ≤ 0.05) to determine independent predictors of the dependent variable.

## Results

Participants’ general characteristics are summarized in (Table 1).

**Table 1:** General characteristics of the participating women

Variables	(*N* = 173) Results
**• Age (years)**	59.6 ± 6.8
**• Age at menopause (years)**	49.7 ± 4.2
**• Years since menopause**	9.8 ± 7.2
**• Age at T2DM diagnosis (years)**	46.7 ± 9.3
**• Years since T2D**	12 (6–20)
**• T2D therapy mode**	
Diet	5 (3 %)
OHD	91 (53 %)
Diet + OHD	3 (2 %)
Insulin	15 (9 %)
Insulin + OHD	59 (34 %)
• **Use of statin therapy**	84 (49 %)
**• Hypertensive** (according to medical records)	
Yes	125 (72 %)
No	48 (28 %)
**• SBP (mmHg)**	144 ± 23
**• DBP (mmHg)**	82 (76–90)
**• Marital status**	
Single	1 (1 %)
Married	113 (65 %)
Divorced	10 (6 %)
Widow	49 (28 %)
**• Education**	
Illiterate	73 (42 %)
Elementary	36 (21 %)
Intermediate	29 (17 %)
Secondary	25 (14 %)
University	10 (6 %)
Postgraduate	0 (0 %)
**• Occupation**	
Housewife	160 (93 %)
Governmental employed	0 (0 %)
Privately employed	4 (2 %)
Self-employed	0 (0 %)
Retired	9 (5 %)
**• Ethnicity**	
White (Arabic)	126 (73 %)
Black (African)	30 (17 %)
South Asian (Pakistani)	17 (10 %)
**• Skin tone (Fitzpatrick)**^a^	
Type I (light, pale white)	0 (0 %)
Type II (white, fair)	24 (14 %)
Type III (medium white to olive)	68 (39 %)
Type IV (olive, mid brown)	50 (29 %)
Type V (brown, dark brown)	31 (18 %)
Type VI (very dark brown, black)	0 (0 %)
**• Sun exposure**	
< 1 h/week	85 (49 %)
1–2 h/week	48 (28 %)
2–3 h/week	21 (12 %)
> 3 h/week	19 (11 %)
**• Veiling type**	
Totally covered (use of niqab: eyes exposed only)	125 (72 %)
Partially covered (face exposed)	48 (28 %)
**• Use of sunscreen**	0 (0 %)
**• Subjects consuming dietary VitD above EAR**^b^	0 (0 %)
**• Physical activity**	
Yes	53 (31 %)
No	120 (69 %)
**• Smoking**	
Yes	3 (2 %)
No	170 (98 %)
**• Serum total cholesterol (mmol/L)**	4.2 ± 1.3
**• Serum triglyceride (mmol/L)**	1.4 (0.99–2.2)
**• Serum HDL-C (mmol/L)**	1.0 (0.8–1.3)
**• Serum LDL-C (mmol/L)**	2.15 (1.7-3.0)
**• Serum VLDL-C (mmol/L)**	0.62 (0.45-1.0)
**• Serum hs-CRP (mg/L)**	5.4 (2.8–9.9)

The numerical data are presented as mean ± SD if normally distributed and as median (IQR) if non-normally distributed. Descriptive data are presented as *n* (%).T2D is Type 2 Diabetes Mellitus. OHD is Oral Hypoglycemic Drugs. *BMI* represents Body Mass Index, *WHR* Waist Hip Ratio, *SBP *Systolic Blood pressure, *DBP* Diastolic Blood Pressure

^a^Fitzpatrick scale [20]. EAR is estimated average requirement

^b^EAR for women aged 50 y and over based on IOM recommendation (600-800 IU/day) [21]. 25(OH)D is 25-hydroxyvitamin D; PTH is Parathyroid Hormone; Ca is Calcium; PO4 is Phosphate; and Mg is Magnesium. HDL-C is high lipoprotein cholesterol; LDL-C is low density lipoprotein cholesterol; VLDL-C is very low density lipoprotein cholesterol; and hs-CRP is high sensitive C-reactive protein

Serum 25(OH)D levels and dietary VitD intake in all participating women and in sub-classified ethnic groups are shown in (Table 2); showing overall serum 25(OH)D mean (± SD) of 14.2 ± 9.2 ng/ml and non-significant differences in median of serum 25(OH) D levels and dietary VitD intake between ethnic groups.

**Table 2:** Serum 25(OH)D levels and dietary VitD daily intake among the participants as classified by ethnicity

Ethnicity	25 (OH)D (ng/ml)	***P***	Dietary VitD intake (IU/day)	***P***
**Overall** (*n*=173)	14.2±9.2		110 (53.5-180)	
**White-**Arabic (*n*=126)	13.1 (7.6-19.2)	0.70	110 (62-168)	0.38
**Black-**African (*n*=30)	10.8 (8.2-17.3)		114 (73-218)	
**Asian-**Pakistani (*n*=17)	12 (6-17.6)		100 (60-176)	

Numerical data are presented as mean ± SD or median (IQR). 25(OH)D is 25-hydroxyvitamin D. Differences in VitD between different ethnic groups were tested by Kruskal-Wallis H test

According to IOM [19] guidelines for VitD status classification, 47 % were VitD deficient, 31 % were VitD insufficient, and 22 % demonstrated optimal levels of VitD.

In the full cohort, the relationship between serum 25 (OH)D level showed an inverse association with body weight (*P* = 0.028), waist and hip circumferences (*P* = 0.033, *P* = 0.004 respectively). Conversely, BMI and WHR did not show any association with total 25 (OH)D (Table 3). When the relationships were investigated within each ethnic group independently, no statistical significant correlation was found except for BMI, which was positively correlated with 25(OH)D in white postmenopausal women (*r*=-0.250, *P* = 0.009, Pearson correlation, 2-tailed).

The correlation between 25(OH)D and bone related parameters were non-significant except for serum intact PTH which showed an inverse relationship with 25(OH)D (*p* < 0.0001) (Table 3). No independent effect of ethnicity was found.

**Table 3:** The serum 25(OH)D correlation with anthropometric measures and bone related parameters in whole group

Variable	Results (***N***=173)	Correlation with 25(OH)D	
		r	***P***
**Weight (kg)**	79.3 ± 18	-0.173*	0.028*
**Height (cm)**	154.7 ± 6	-0.020	>0.1
**BMI (kg/m²)**^**(a)**^	49.5 (43.3-58)	-0.120	>0.1
**Waist circumference (cm)**	100.2 ± 12.7	-0.167*	0.033*
**Hip circumference (cm)**	113.5 ± 13.4	-0.220*	0.004*
**WHR**	0.9 ± 0.06	0.086	>0.1
**Serum Intact PTH (pg/ml)**^**(a)**^	47.9 (33.3-61.9)	-0.340*	<0.0001*
**Serum Ca (mmol/L)**^**(a)**^	2.25 (2.07-2.42)	0.009	>0.1
**Serum PO**_**4**_**(mmol/L)**	1.19±0.2	-0.060	>0.1
**Serum Mg (mmol/L)**^**(a)**^	0.7 (0.6-0.8)	-0.110	>0.1

Results are presented as mean ±SD or median (IQR)

*PTH* parathyroid hormone, *Ca* calcium, *PO4* phosphorus, *Mg* magnesium

^a^Spearman Correlation (2-tailed). The rest of correlations are Pearson correlations (2-tailed)

*Significant correlation (*p*<0.05)

**Fig. 2:**
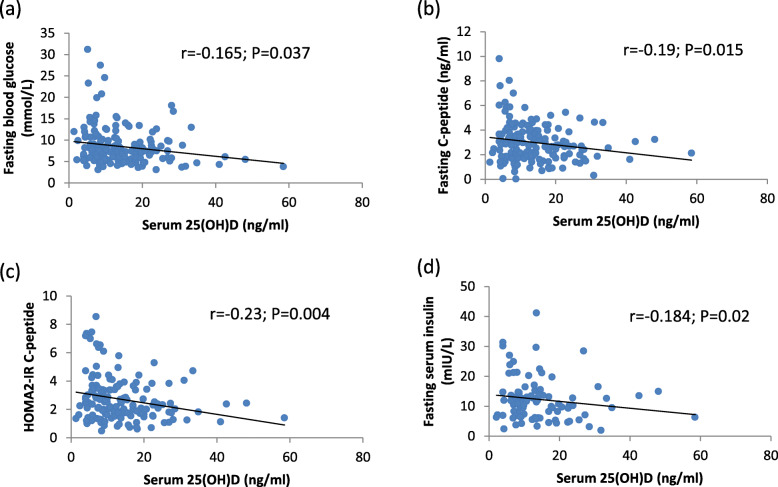
The relationship between 25(OH)D and glycaemic parameters. **a** The relationship between total 25(OH)D and fasting glucose (*n* = 173,2-tailed Spearmen correlation). **b** The relationship between total 25(OH)D and fasting C-peptide (*n* = 173, 2-tailed Pearson correlation). **c** The relationship between total 25(OH)D and HOMA2-IR C-peptide (*n* = 173, 2-tailed Spearmen correlation). **d** The relationship between total 25(OH)D and fasting insulin (*n* = 99*, 2-tailed Pearson correlation). *Subjects not taking insulin

When correlations were assessed between serum 25(OH)D and the glycaemic control parameters, a significant negative correlation was found between 25(OH)D and: fasting glucose (*P* = 0.037), fasting insulin (*P* = 0.02) fasting C-peptide (*P* = 0.015), HOMA2- IR C-peptide (*P* = 0.004) (Fig. 2). The correlations between 25(OH) and the remaining glycaemic control parameters (including HbA1c, HOMA2- IR insulin, HOMA2-%β**)** were not significant.

After sub-dividing the group according to ethnicity, 25(OH)D was associated with serum fasting C-peptide and HOMA2-IR C-peptide in white group only, while it was not associated with any parameters in either black and Asian groups (Table 4). Additionally, 25(OH)D levels did not show any significant correlation with skin tones, sun exposure, dietary VitD intake, veiling type, age, duration of diabetes and menopause, ethnicity, presence of hypertension, diabetes treatment, BMI class, smoking, marital status, occupation or education (data not shown).

**Table 4:** Correlations between 25(OH) D and glycaemic parameters among different ethnic groups of the study participants

Variable	Results (***N***=173)	White (***n***=126)		Black (***n***=30)		Asian (***n***=17)	
		r	***P***	r	***P***	r	***P***
**Fasting insulin (pmol/L)**^**a**^	93.05 ± 72.9	-0.14	>0.1	-0.27	0.27	-0.24	>0.1
**Fasting c-peptide (nmol/L)**	0.98 ± 0.51	-0.23*	0.012*	-0.03	0.86	-0.21	>0.1
**Fasting glucose (mmol/L)**	7.4 (5.6-10.2)	-0.17	0.065	-0.13	0.57	0.075	>0.1
**HbA1c (mmol/mol)**	64 ± 864 ± 8	-0.03	>0.1	-0.21	0.29	0.003	>0.1
**HOMA2-IR insulin**^**a**^	2.69 ± 1.54	0.20	>0.1	-0.23	0.35	-0.20	>0.1
**HOMA2-%β insulin**^**a**^	79.9 (46.1-136.9)	0.10	>0.1	0.045	0.86	-0.10	>0.1
**HOMA2-IR C-peptide**	2.7 ± 8.56	-0.25*	0.009*	-0.23	0.27	-0.26	>0.1
**HOMA2-%β C-peptide**	69.5 (35.3-102.6)	0.066	>0.1	0.12	0.57	-0.23	>0.1

Correlations in white group are Pearson correlation (2-tailed). Correlations in black and Asian group are Spearman correlations (2-tailed)

^a^Measured in subjects not taking exogenous insulin, total (*n*=99): white (*n*=68), black (*n*=21) and Asian (*n*=10). HOMA2-IR is homeostatic assessment 2 for insulin resistance. HOMA2-%β is homeostatic assessment 2 for β-cell function; HOMA2-IR/% β C-peptide was calculated using fasting glucose and C-peptide; HOMA2- IR/% β insulin was calculated using fasting glucose and fasting insulin

^*^Significant correlation (*p*<0.05)

Multiple linear regression analysis results for the study subjects are shown in (Table 5). Hip circumference and HOMA2-IR C-peptide were the only independent variables showing statistical significance as predictors for serum 25(OH)D levels, explaining 8.5 % of variability in serum vitD levels.

**Table 5:** Stepwise multiple regression analysis between serum 25(OH)D level and independent variables (*N*=173)

Dependent variable	Independent variables	β	***p***	95% CI for β	
				lower limit	upper limit
Serum 25(OH)D	Hip circumference (cm)	-0.150	0.007	-0.258	-0.041
	HOMA2-IR C-peptide	-1.252	0.008	-2.175	-0.328
**Total R**^**2**^**= 0.085**					

Stepwise variable inclusion with *p* < 0.05 and exclusion with *p* > 0.10

*95% CI* confidence intervals, *ß* unstandardized regression coefficient, *R*^*2*^ percent variance explained by each variable

## Discussion

Prevalence of VitD deficiency between 40 and 100 % were previously identified in studies in elderly US and European cohorts [25–36]. In the Middle East including Saudi Arabia, 25(OH)D deficiency has also been documented previously despite the abundance of sunlight, with almost half of the study subjects (47 %) having VitD deficiency and 31 % VitD insufficiency. This high prevalence of VitD deficiency or insufficiency found in our study subjects was as expected due to several factors influencing negatively on VitD status including lack of adequate sunlight exposure (due to veiling and extreme hot weather) and inadequate dietary VitD intake.

The current study is the first to investigate insulin resistance and sensitivity (including HOMA-2) in multi-ethnic groups in Saudi Arabia (Jeddah). VitD in this study was found to be significantly correlated (p < 0.05) with fasting insulin, fasting glucose, fasting C-peptide and insulin resistance indices (HOMA2-IR C-peptide). This finding was consistent with what was reported by Forouhi et al. [37], Hahn et al. [38], Weiler et al. [39] and Dutta et al. [40]. These associations between VitD and glycaemic parameters in our study can be attributed to the biological mechanism whereby VitD has a direct effect on the pancreatic β-cell by binding to VDR or an indirect effect through its role in regulation of extracellular Ca and Ca flux into the pancreatic β-cell [41]. Our results show that ethnicity can modify the associations between VitD and glycaemic markers as 25(OH)D was associated with serum fasting C-peptide and HOMA2-IR C-peptide in the white group (*n* = 126), but not associated with any diabetic parameter in either the black (*n* = 30) and Asian (*n* = 17) groups. This finding confirms results from a large cross-sectional US study in non-Hispanic whites and blacks carried by Scragg et al., where 25(OH)D was associated with HOMA-IR in whites but not in blacks [14]. The mechanism underlying this absence of an association between 25(OH)D and insulin resistance in blacks is unclear. This observed differences in the relationship between VitD and T2D between blacks and whites might be due to the variation in the threshold at which VitD takes effect in different ethnicities and a possible decreased responsiveness to VitD and PTH in blacks [14]. In addition, ethnic differences in factors such as culture, genetic background, lifestyle and diet might also contribute to the variation in the VitD relationship with T2D. Further studies are needed to clarify this ethnic discrepancy in VitD action, which could only be achieved using a supplementation protocol, thus providing novel insight into potential preventive mechanisms linked to VitD for these specific groups.

Our data failed to show any association between VitD and glycemic control (HbA1c) which was in line with what has been reported [42, 43]. Moreover, a meta-analysis of fifteen dietary intervention trials demonstrated that, in patients with type 1 and 2 diabetes (or patients with impaired glucose intolerance), VitD had no impact on reducing HbA1c [44]. However, the situation is far from clear, with several studies finding an association with HbA1c, including a cohort study from Saudi Arabia in 1000 patients with type1 and 2 diabetes which demonstrated an inverse correlation [45–48]. Diabetes is a heterogenous disease with multiple treatment modalities and so discrepancies between studies are to be expected, a problem which can only be addressed by more defined and larger populations within studies.

In the present study, we explored VitD association with measures of obesity in T2D and we reported that VitD was inversely related to weight, waist and hip circumferences. This is not unanticipated, as obesity has an adverse effect on VitD status due to of 25(OH)D being sequestered within adipose tissue [49, 50]. However, VitD was not related to BMI or WHR (conventional measures of overall obesity and central obesity respectively), which is in contrast with what some other studies have observed [49, 51, 52]. However, when ethnicity was taken into account, 25(OH)D was associated with BMI in white women. In comparison, 25(OH)D was not associated with anthropometric measures in the other two ethnic groups (black and Asian). These findings are in agreement with findings of other studies that has shown that ethnicity might modify the relationship between adiposity (including BMI and WHR) and serum 25(OH)D. In prior studies, either a lack of association has been observed between these variables in single multi-ethnic study groups or, different associations are found between different ethnicites [14, 53, 54]. These observations question whether VitD supplementation will have the same effect on obesity and insulin resistance among individuals from different ethnic groups. This also highlights a future need to investigate anthropometric and glycaemic responsiveness to VitD supplementation in multiple ethnicities, and could suggests ethnically personalized VitD recommendations against obesity and/or insulin resistance.

Recently, several studies have examined the relationship between 25(OH)D, obesity and diabetes, reporting lower 25(OH)D [55, 56]. Obesity is considered as a primary risk factor for T2D and so it challenging to disentangle whether low 25(OH)D is related directly to T2D, or indirectly through obesity [56–58]. In the present study, waist circumference and HOMA2-IR C-peptide remained predictors of vitD status after multiple regressions supporting vitD association with obesity and insulin resistance.

Overall, there is a need for postmenopausal women with T2D in Saudi Arabia to increase their VitD levels (which can be approached by economical approaches including VitD supplementation and increasing sunlight exposure). In addition, further studies are required to explore potential VitD protective mechanisms for T2D and adiposity in various ethnic cohorts, to understand observed disparity of VitD impact between different ethnicities. Derivation of an appropriate cut-off level for 25(OH)D for improving insulin resistance would need to be considered in the future study.

The present study has several limitations that have to be taken into account or consideration when interpreting the results. Sample sizes of subcategorized black and Asian ethnic groups were small and would need to be increased in order to confirm these findings related to ethnicity. Another limitation is that this study is cross-sectional in nature, therefore, causality of temporal VitD associations in T2D cannot be confirmed. Furthermore, variations in diabetic treatment regimens and duration of T2D amongst participating women might contribute to the VitD relationship with glycaemic control parameters. Physical activity and VitD dietary intake were both assessed by self-report. Although, the present study relied on single measurements of vitD and did not consider seasonal variation of vitD level, the abundance of sunshine throughout the years in Saudi Arabia makes the seasonal aspect less relevant than in other cohorts.

## Conclusions

VitD deficiency (serum 25(OH)D < 12 ng/ml) is highly prevalent among postmenopausal women with T2D. Our findings confirm the inverse VitD relationship with some measures of obesity and insulin sensitivity in T2D, however this association was only observed in white subjects but not in those from black or Asian origin. Further studies are required to understand the underlying mechanism responsible for this ethnic variation and to explore and compare VitD supplementation responses in black, and white and Asian ethnic groups.

## Data Availability

The datasets used and/or analysed during the current study are available from the corresponding author on reasonable request.

## Abbreviations

25(OH)D: 25-hydoxyvitamin D
ALP: Alkaline phosphatase
ALT: Alanine aminotransferase
AST: Aspartate aminotransferase
Ca: Calcium
CEOR: Centre of Excellence for Osteoporosis Research
CLIA: Competitive chemiluminescence immunoassay
CV: Coefficient of variation
FSH: Follicular stimulating hormone
Hb: Hemoglobin
HbA1c: Glycosylated hemoglobin percentage
HDL-C: High density lipoprotein cholesterol
HEGC: Hyperinsulinemic euglycemic clamp
HOMA-%β: Homeostatic model assessment β-cell secretion
HOMA-IR: Homeostatic model assessment of insulin resistance
Hs-CRP: High sensitive C-reactive protein
IQR: Interquartile range
KAU: King Abdul-Aziz University
KAUH: King Abdul-Aziz University Hospital
KFMRC: King Fahad Medical Research Centre
LDL-C: Low density lipoprotein cholesterol
LMP: Last menstrual period
Mg: Magnesium
PHCC: Primary health care center
PO4: Phosphate
PTH: Parathyroid hormone
QUICK-I: Quantitative insulin sensitivity check index
T2D: Type 2 diabetes mellitus
T3: Thyroxin
T4: Triiodothyronine
TFT: Thyroid function test
TSH: Thyroid-stimulating hormone
VDBP: Vitamin D binding protein
VitD: Vitamin D
VLDL-C: Very low density lipoprotein cholesterol
WC: Waist circumference
WHR: Waist-hip ratio
